# Enhanced cell disruption strategy in the release of recombinant hepatitis B surface antigen from *Pichia pastoris* using response surface methodology

**DOI:** 10.1186/1472-6750-12-70

**Published:** 2012-10-05

**Authors:** Yew Joon Tam, Zeenathul Nazariah Allaudin, Mohd Azmi Mohd Lila, Abdul Rani Bahaman, Joo Shun Tan, Morvarid Akhavan Rezaei

**Affiliations:** 1 Department of Veterinary Pathology and Microbiology, Faculty of Veterinary Medicine, Universiti Putra Malaysia, Serdang, 43400, Selangor, Malaysia; 2 Laboratory of Immunotherapeutic and Vaccine Technology (LIVES), Institute of Bioscience, Universiti Putra Malaysia, Serdang, Selangor, Malaysia

**Keywords:** Hepatitis B surface antigen, Cell disruption, Glass bead, High pressure homogenizer, *Pichia pastoris*, Recombinant protein

## Abstract

**Background:**

Cell disruption strategies by high pressure homogenizer for the release of recombinant Hepatitis B surface antigen (HBsAg) from *Pichia pastoris* expression cells were optimized using response surface methodology (RSM) based on the central composite design (CCD). The factors studied include number of passes, biomass concentration and pulse pressure. Polynomial models were used to correlate the above mentioned factors to project the cell disruption capability and specific protein release of HBsAg from *P. pastoris* cells.

**Results:**

The proposed cell disruption strategy consisted of a number of passes set at 20 times, biomass concentration of 7.70 g/L of dry cell weight (DCW) and pulse pressure at 1,029 bar. The optimized cell disruption strategy was shown to increase cell disruption efficiency by 2-fold and 4-fold for specific protein release of HBsAg when compared to glass bead method yielding 75.68% cell disruption rate (CDR) and HBsAg concentration of 29.20 mg/L respectively.

**Conclusions:**

The model equation generated from RSM on cell disruption of *P. pastoris* was found adequate to determine the significant factors and its interactions among the process variables and the optimum conditions in releasing HBsAg when validated against a glass bead cell disruption method. The findings from the study can open up a promising strategy for better recovery of HBsAg recombinant protein during downstream processing.

## Background

The worldwide importance of hepatitis B virus (HBV) infection and the toll it takes in chronic liver disease, cirrhosis and hepatocellular cancer is one of the major causes of the global death affecting more than 2 billion people [1]. Since the introduction of hepatitis B vaccine two decades ago, immunization has been made available to several million individuals as an effective means of preventing hepatitis B associated health problems [2]. However, there are still more than 400 million chronic carriers, with the risk of hepatitis B infection on the rise due to globalization that had radically changed the way transmissible viral diseases shape their epidemiology [3,4]. To date, there is no effective treatment for acute hepatitis B and the only means of prevention is through vaccination. More than 90 countries are now implementing universal vaccination to new-borns against HBV [5]. Indeed, small HBsAg consisting of 226 amino acids that assembles into 20–22 nm particles, is antigenically most significant from vaccine development point of view [6,7]. As such, the HBsAg gene has been successfully expressed in a variety of hosts such as prokaryotic organisms [8], yeasts [9-11], mammalian cells [12] and plants [2,13]. Subsequently, methods of releasing product from cells following fermentation will be required. For isolation of intracellular biotechnology products, the operation of cell disruption is considered a critical step in product recovery from host microorganism which usually is essential for downstream processing since this step influences the quantity of the desired protein, the ease of subsequent purification steps as well as its biological activity [14]. In the production of recombinant proteins, the ratio of variable recovery process costs to fermentation costs vary from 1 to 3 for enzyme and antibiotic recovery, and the ratio reached up to 10 for the recovery of intracellular recombinant insulin [15,16]. Optimization of cell disruption process would therefore be economically important in achieving efficient and cost effective product recovery [17].

Considerable attention was given to high-pressure homogenization (a mechanical disruption technique) that is the most widely used method for process-scale cell disruption [18]. High pressure homogenization cell suspensions are pressurized by positive displacement pump and passed through a minute gap in the valve and impactor arrangement to disintegrate the cell. The different valve characteristics and impactor arrangement yield different performances of cell disruption for different microorganisms [16]. Ideally, efficient cell disruption can be achieved by ensuring maximum product release while at the same time limiting the exposure to severe conditions in a homogenizer to minimise product denaturation and excessive formation of small cell debris fragments. The latter can have critically and detrimentally effect on subsequent downstream product recovery and purification [15,16]. The mechanism of cell disruption in high-pressure flow devices, such as the APV Manton-Gaulin homogenizer, has been the focus for cell disruption processes [16,19-21]. Details design of valve and impactor arrangement and its effect on the disruption of various microorganisms have been discussed and reviewed in previous literatures [22-24].

Similarly, a small-scale Avestin homogenizer (Emulsiflex-C50, Canada) that was initially used in emulsion industries has been adapted to be used for cell disruption [25-27]. The Avestin homogenizer can be operated at a pressure up to 2,000 bar and can accommodate a wide range of sample volumes (0.05-50 L per hour) depending on pressure setting. The system would be suitable for scale-down operations to enable initial generation of reliable data for process scale-up using small quantities of material that offers the potential for rapid screening of process options and for the acceleration of the design phase before scale-up implementation with concomitant reduction in development costs and time [28]. The performance of homogenizer is influenced by a number of parameters such as pulse pressure, cell concentration, cell growth conditions, feed flow rate, number of passes and type of micro-organism on were also shown to have effects on the extent of product recovery and the quality of the final product [23,24,29].

Conventional practice of single factor optimization by maintaining other factors at an unspecified constant level does not depict the combined effect of all the factors involved. Optimization of parameters by the conventional method involves changing one independent variable while unchanging all others at a fixed level. This is extremely time-consuming and expensive for a large number of variables [30] and also may result in wrong conclusions [31]. On the other hand, response surface methodology (RSM) is one of the strong tools to know the effect of many factors with less number of experiments and can also be used to refine the optimization of the process. It defines the effects of each variable independently, as well as the contribution of joint effects of variables, which cannot be observed by traditional optimization methods [32]. It has been applied for the optimization for medium and cultural conditions in bioprocesses [31,33].

Therefore, in the present study, RSM based on a central composite design (CCD) was used to identify main factors (number of passes, biomass concentration and pulse pressure) influencing cell disruption capability of Avestin homogenizer on *P. pastoris* and to optimize the cell disruption process in maximizing the recovery of recombinant HBsAg. The adaptation of the optimized conditions for higher volume processing is also demonstrated.

## Results

### CCD experimental run and statistical analysis

The optimization of the high pressure homogenizer cell disruption process was carried out to find the optimal values of independent factors (number of passes, cell concentration, and pulse pressure), which provides efficient cell disruption and maximum release of HBsAg. RSM based on the use of CCD was performed with results obtained from each experimental run examined as described in the design matrix (Table 1). From the table, experimental runs 5–10 and 14–20 showed high capability of cell disruption with high percentage of CDR (> 70%) after passing through the high pressure homogenizer, which ranges from 70.04% to 78.96%. The highest disruption capability was achieved at CDR 78.96% of the amount of cells disrupted in run 20 and the lowest capability of cell disruption was observed in run 3 with CDR of 27.64%. For specific protein release, experimental runs 15–20 showed the highest level of HBsAg released with the concentration ranging from 28.62 to 31.63 mg/L. The maximum HBsAg released was achieved in run 19 (31.63 mg/L), while minimum HBsAg released was observed in run 13 (3.68 mg/L) showing close similarity with the predicted results. In general, cell disruption capability was seen to perform well in the mid-level of each factor while the highest specific protein release was obtained under the same conditions when number of passes, biomass concentration and pulse pressure were simultaneously increased to 20 times, 7.70 g/L and 1,000 bar respectively. The results also demonstrated that cell disruption capability shares close relationship with the release of specific protein to a certain extent (Table 1). For example, when high cell disruption capabilities were observed in experimental runs 15–20 (CDR >70%), high concentration of HBsAg (> 28 mg/L) were obtained. Vice versa, in experimental runs 1–4 and 13, low cell disruption capability (CDR <55%) results in low recovery of HBsAg. However, although high cell disruption capabilities was observed in experimental runs 5–10, lower levels of HBsAg concentration were detected (8.65-15.10 mg/L). Thus, the possibility of using a linear description on the correlation between the two factors was omitted.

**Table 1:** Full factorial CCD matrix for the three significant variables and experimental and predicted values of cell disruption capability measured by CDR and specific protein release measured by ELISA for HBsAg concentration

	**Variables**			**Cell disruption capability**		**Specific protein**	
**Experimental runs**	**Number of passes (times)**	**Biomass concentration (g/L)**	**Pulse pressure (bar)**	**CDR (%)**		**HBsAg (mg/L)**	
				**Experimental**	**Predicted**	**Experimental**	**Predicted**
1	15(−1)	5.74(−1)	600(−1)	53.83	56.04	8.74	8.96
2	25(+1)	5.74(−1)	600(−1)	41.17	46.21	6.74	5.12
3	15(−1)	9.65(+1)	600(−1)	27.64	37.44	10.99	9.92
4	25(+1)	9.65(+1)	600(−1)	31.8	38.05	7.55	6.05
5	15(−1)	5.74(−1)	1400(+1)	76.7	76.9	8.65	9.53
6	25(+1)	5.74(−1)	1400(+1)	74.27	70.92	8.89	9.34
7	15(−1)	9.65(+1)	1400(+1)	74.69	76.11	9.46	10.47
8	25(+1)	9.65(+1)	1400(+1)	76.31	80.56	11.09	10.25
9	12(−α)	7.70(0)	1000(0)	75.41	70.42	15.1	14.18
10	28(+α)	7.70(0)	1000(0)	70.04	65.9	8.98	10.77
11	20(0)	4.41(−α)	1000(0)	65.77	66.45	8.98	8.72
12	20(0)	10.98(+α)	1000(0)	68.72	58.91	9.17	10.3
13	20(0)	7.70(0)	327.28(−α)	36.75	26.01	3.68	5.74
14	20(0)	7.70(0)	1672.72(+α)	77.68	79.3	10.94	9.75
15	20(0)	7.70(0)	1000(0)	74.57	75.74	30.25	29.75
16	20(0)	7.70(0)	1000(0)	72.24	75.74	29.39	29.75
17	20(0)	7.70(0)	1000(0)	75.2	75.74	30.48	29.75
18	20(0)	7.70(0)	1000(0)	75.62	75.74	28.29	29.75
19	20(0)	7.70(0)	1000(0)	76.55	75.74	31.63	29.75
20	20(0)	7.70(0)	1000(0)	78.69	75.74	28.62	29.75

() represents coded values.

**Figure 1:**
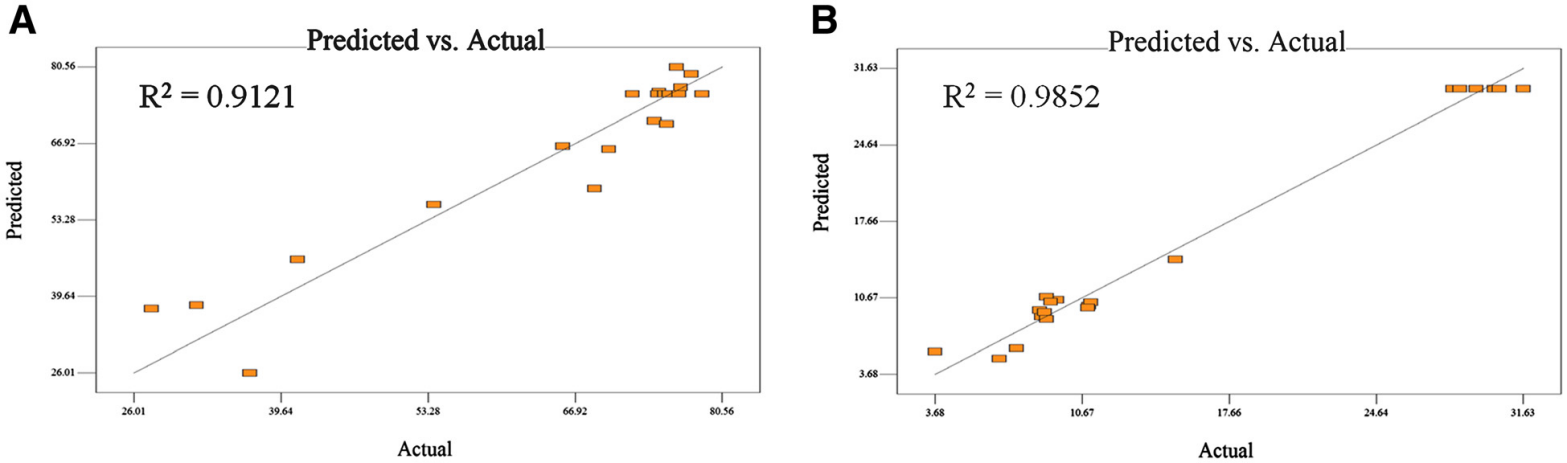
**Plot indicating the predicted values against experimental values for cell disruption capability (A) and specific protein release of HBsAg (B).** The linear line depicted represents y=x.

The independent factors were fitted to the second order-model equation and examined for the goodness of fit. The significance of the model was validated with the predicted optimum values by the applied equation and experimental cell disruption capability and specific protein release of HBsAg (Figure 1 A and B). The regression equation obtained indicated the R^2^ value of 0.9121 for cell disruption capability and 0.9852 for specific protein release (a value of R^2^ greater than 0.75 indicates the aptness of the model). This value ensured a satisfactory adjustment of quadratic model to the experimental data and indicated that the theoretical values as predicted by the models fitted well to the experimental data (Figure 1).

The results of second order response surface model in the form of analysis of variance (ANOVA) are given in Table 2. Fisher’s F-test and *p*-values demonstrate significance for the regression model for both cell disruption capability and specific protein release. The ANOVA table indicates the overall significant effect of interaction terms on HBsAg release using high pressure homogenizer at 5% level of significance (*p*<0.005) and F calculated > F table. Low pure errors for cell disruption capability (22.87) and specific protein release (7.87) indicate a good reproducibility of the experimental data. The F-value for both cell disruption capability (11.53) and specific protein release (74.01) indicates that the model is significant. There is only a 0.03% and 0.01% chance that the model F-value this large could occur due to noise respectively.

**Table 2:** Analysis of Variance (ANOVA) for quadratic model on the cell disruption capability and specific protein release activity of HBsAg from the result of CCD

**Source**	**DF**	**Sum of Squares**	**Mean Square**	**F value**	***p***
**Cell disruption capability**					
Model	9	4946.52	549.61	11.53	0.0003
A	1	24.63	24.63	0.52	0.4887
B	1	68.42	68.42	1.44	0.2586
C	1	3427.89	3427.89	71.9	< 0.0001
A^2^	1	103.49	103.49	2.17	0.1714
B^2^	1	307.23	307.23	6.44	0.0294
C^2^	1	960.37	960.37	20.14	0.0012
AB	1	54.44	54.44	1.14	0.3104
AC	1	7.39	7.39	0.16	0.702
BC	1	158.33	158.33	3.32	0.0984
Residual	10	476.79	47.68		
Lack of Fit	5	453.91	90.78	19.85	0.0026
Pure Error	5	22.87	4.57		
**Specific protein**					
Model	9	1841	204.56	74.01	< 0.0001
A	1	14.09	14.09	5.1	0.0476
B	1	2.99	2.99	1.08	0.3228
C	1	19.4	19.4	7.02	0.0243
A^2^	1	537.7	537.7	194.56	< 0.0001
B^2^	1	737.91	737.91	267	< 0.0001
C^2^	1	872.46	872.46	315.68	< 0.0001
AB	1	2.85E-04	2.85E-04	1.03E-04	0.9921
AC	1	6.68	6.68	2.42	0.1511
BC	1	2.85E-04	2.85E-04	1.03E-04	0.9921
Residual	10	27.64	2.76		
Lack of Fit	5	19.77	3.95	2.51	0.1675
Pure Error	5	7.87	1.57		

The estimated parameter and the corresponding *p*-values suggested that, for independent factors, pulse pressure had significant effects on both cell disruption capability and specific protein release of HBsAg from *P. pastoris* via cell disruption process. It is evident that pulse pressure was a key factor influencing the cell disruption process owing to the lowest *p*-value among the others (Table 2). For specific protein released, number of passes was also determined to have significant effect as indicated by low *p*-value. However, it was observed to be less significant when compared to pulse pressure. After neglecting the effect of insignificant interacting terms from the general quadratic model, the simplified quadratic models for the cell disruption capability and specific protein release of HBsAg protein by cell disruption using high pressure homogenizer were constructed in terms of coded values and are shown in Equations 1 and 2 respectively.

(1) $$\text{Y}=75.74+15.84\text{C}–4.{62\text{B}}^{2}–8.{16\text{C}}^{2}$$

(2) $$\begin{array}{ll} \text{Y} & =29.75–1.02\text{A}\\ & \;+1.19\text{C}–6.{11\text{A}}^{2}–7.{16\text{B}}^{2}–7.{78\text{C}}^{2} \end{array}$$

### Effect of interaction factors on cell disruption capability by high pressure homogenizer and specific protein release of HBsAg protein from *P. pastoris* by cell disruption

**Figure 2:**
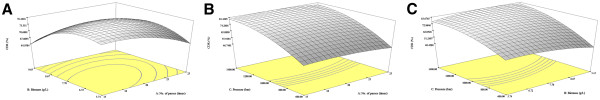
**Response surface 3D contour plots for effect of three main variables on cell disruption capability as (A) Number of passes and Biomass concentration; (B) Number of passes and Pulse pressure; and (C) Biomass concentration and Pulse pressure.** When the effect of two variables was plotted, the other factor was set at middle level point.

**Figure 3:**
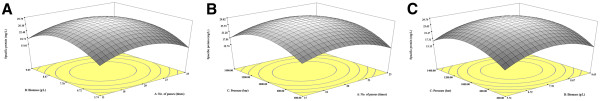
**Response surface 3D contour plots of the combined effects of (A) Number of passes and Biomass concentration; (B) Number of passes and Pulse pressure and (C) Biomass concentration and Pulse pressure on specific protein release of HBsAg from cell disruption of*P. pastoris*cells.**

Three-dimensional response surface and contour plots described by the regression model were drawn to illustrate the effects of the independent factors and the interactive effects of each independent factor on the response factors. The plots were generated for the response at any two independent factors while keeping the others at the middle level (level 0) shown in Table 3. The shape of the corresponding response surfaces and contour plots indicates the interactive responses of the factors. The significant interaction factors for cell disruption capability and specific protein release of HBsAg are shown in Figures 2 and 3.

**Table 3:** Values of independent variables at different levels of CCD design

**Independent variable**	**Levels**				
	-α (1.682)	−1	0	+1	+α (1.682)
**Number of passes (times)**	12	15	20	25	28
**Biomass concentration (g/L)**	4.41	5.74	7.70	9.65	10.98
**Pulse pressure (bar)**	327.28	600	1,000	1,400	1672.72

Figure 2A depicts a three-dimensional response surfaces showing the effects of number of passes and biomass concentration on cell disruption capability while pulse pressure was fixed at the middle level (1,000 bar). The elliptical nature of the contour plot and its three-dimensional response surface plot indicated that the interaction between A and B were significant. CDR was observed to increase with the increase in number of passes creating a range of 15 to 20 times with respect to pulse pressure and at biomass concentration between 5.74-7.70 g/L (Dry cell weight (DCW)). Down-slope pattern was then observed with the decrease of CDR for both number of passes beyond 20 times onwards and at biomass concentration above 7.70 g/L. Figure 2B shows the effect of pulse pressure and number of passes on cell disruption capability where the biomass concentration is fixed at the middle level (7.70 g/L). Increase in response was obtained upon increasing pulse pressure. Under the influence of pulse pressure factor, number of passes was observed to have no significant effect on cell disruption capability under the range of 15 to 25 times. The effect of pulse pressure in combination with effect of biomass was seen in line with increased cell disruption capability in the increase of pulse pressure up to 1,000 bar. Increased pulse pressure beyond 1,000 bar generates shows no significance in cell disruption capability indicating that maximum cell disintegration was achieved (Figure 2C). Here, the increase of biomass concentration (5.74-9.65 g/L) produces little effect with slight declination in CDR.

Response surface plot for specific protein release of HBsAg towards interaction of biomass concentration and number of passes as variables with pulse pressure at 1,000 bar as central point indicates a response surface plot in a mountain-shaped pattern. The plot indicated a progressive increase in the release of HBsAg up to 7.70 g/L and 20 passes (Figure 3A). Thereon, the increase in biomass concentration to 9.65 g/L and number of passes to 25 times demonstrated a gradual decrease in the release of specific protein at a constant value of 1,000 bar. Similar to Figure 3A, the response surface plot with a constant biomass concentration showed a surface with a maximum stationary point, in which the release of specific protein increased from an initial pulse pressure from 600 bar with number of passes 15 times up to a certain level. The response then decreased with further increments in both pulse pressure and number of passes (Figure 3B). Other interactive effects of pulse pressure and biomass concentration can also be seen in Figure 3C where number of passes was kept constant. Figure 3C demonstrated a resemblance on the outcome of specific protein release in regards to the interactive effects of pulse pressure and biomass concentration at central value of number of passes at 20 times. Release of specific HBsAg protein was found favourable at the optimum pulse pressure (1,000 bar) and a decrease in specific protein release of HBsAg thereafter in relative to increase in biomass. Hence, it was observed that the range of pulse pressure for maximum specific protein release of HBsAg lies between 800 bar to 1,200 bar while the number of passes lies between 18 times to 23 times and the biomass concentration lies between 6.72-8.67 g/L. For combinative effects of the variables, interactions between number of passes and pulse pressure was found to be more significant compared to the other two interactions as shown in *p*-values in Table 2.

### Effect of three variables in cell disruption process of *P. pastoris* cells corresponding to the response of total soluble protein released and selective product recovery

**Figure 4:**
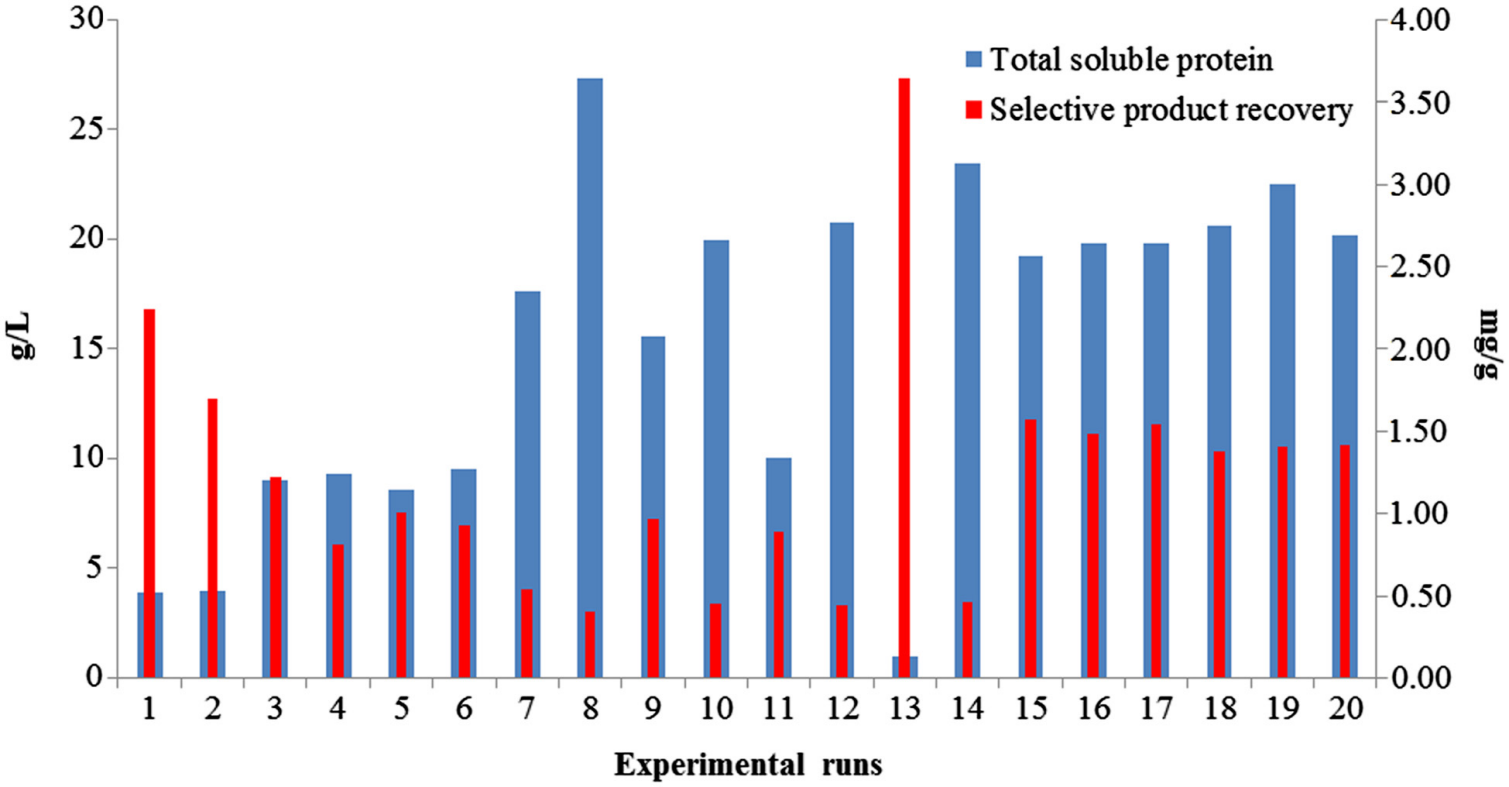
**Total soluble protein (g/L) and selective product recovery (mg/g) of cell disruption process by the effects of three variables.** Total soluble protein was measured using Bradford assay and selective product recovery was calculated based on the ratio of specific protein concentration measured by ELISA method to total soluble protein released. Experimental runs here mentioned are referred as in Table 1.

**Figure 5:**
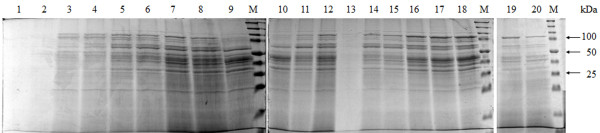
**SDS PAGE of disrupted samples derived from various stages of homogenizer.** Lane 1–20 represents the experimental runs as depicted in Table 1 and lane M represents broad range protein marker.

Results show high total soluble protein was released from *P. pastoris* cells by high pressure homogenizer could be achieved above low pressure range (≤600 bar) as displayed in Figure 4. In experimental runs 1–4 and 13, total soluble protein released was observed to be lower than that of higher pressure range although the effect of biomass concentration produces differences of total soluble protein released between 5.74 g/L and 9.65 g/L of the samples. Increase in number of passes to 25 times showed little significance to the release of total soluble protein at this range. At the high pulse pressure range (≥1,400 bar) for experimental runs 5–8 and 14, maximum release of total soluble protein was seen in experimental run 8 with 27.34 g/L. In this range, increment of number of passes and biomass concentration both shows significant differences on the amount of total soluble protein released. For selective product recovery, high ratio was observed at experimental runs 1–2 and 13 (1.70-3.64 mg/g) with highest selective product recovery in experimental run 13 (3.64 mg/g) at pulse pressure of 327.28 bar while lower selective product recovery (0.41-0.54 mg/g) was seen in experimental runs 7–8, 10 and 12 with the lowest ratio to be in experimental run 8 (0.41 mg/g). Overlapping of the selective product recovery to total soluble protein release demonstrated that generally high selective product recovery in contrast to total soluble protein was obtained in low pulse pressure range and vice versa in the high pulse pressure range (experimental run 12 and 13). However, the extent of the pulse pressure effect were also influenced by other variables as seen in experimental runs 3–4 in low pressure range where the increase in biomass concentration at this range was shown to produce a lower selective product recovery to total soluble protein relative to experimental runs 1–2. Likewise, in high pressure range, increased biomass concentration produced a similar pattern between experimental runs 5–6 and 7–8. The effect of number of passes was also observed to play an important role towards cell disruption where the increased in number of passes to 25 times was shown to produce lower ratio of selective product recovery in both low and high pressure ranges. SDS PAGE analysis (Figure 5) shows that the profiles of experimental runs conducted with determined parameters by RSM (Table 1).

### Optimization of cell disruption process

In order to optimize the process conditions for cell disruption of *P. pastoris* cells for the release of HBsAg by numerical optimization technique, equal importance of ‘3’ was given to all the three processes parameters (number of passes, biomass concentration and pulse pressure) and response (cell disruption capability and specific protein release of HBsAg). The main criterion for constraints optimization was maximum possible release of HBsAg specific protein. The optimum operating conditions for the cell disruption process to achieve maximum specific protein release of HBsAg were 20 times number of passes, 7.76 g/L of biomass concentration and pulse pressure at 1,029 bar. At these conditions of process variables the predicted value of cell disruption capability was at 76.86% CDR with specific protein released to be 29.84 mg/L. The results of optimization were validated by conducting the experiments in triplicates at the above optimized values with ± 1.90 and ± 0.96 standard deviation for cell disruption capability (mean value of 75.68%) and specific protein release of HBsAg values (mean value of 29.20 mg/L) displayed in Table 4. In validation result, the experimental values closely agrees with the value obtained from the RSM, confirming that the RSM using the CCD statistical design of experiments could be effectively used to optimize the process parameters and to study the importance of individual, cumulative and interactive effects of the test variables. Results obtained from optimal cell disruption strategy using RSM for cell disruption capability and for the release of specific protein was increased 2-fold and 4-fold as compared with those obtained with glass bead cell disruption.

**Table 4:** Cell disruption conditions predicted by RSM and validation of the experimental data for cell disruption capability and specific protein release of HBsAg by optimal cell disruption strategy

	**Optimal cell disruption strategy**	**Glass bead cell disruption**
**Parameters**		
Biomass concentration (g/L)	7.76	7.70
Number of passes (times)	20	-
Pulse pressure (bar)	1,029	-
Vortexing (times)	-	20
Glass beads (g/L)	-	40
**Results**		
CDR (%)	75.68	37.18
Specific protein (mg/L)	29.20 ± 0.96	7.28 ± 1.06

### Scale-up of the optimized cell disruption process

**Figure 6:**
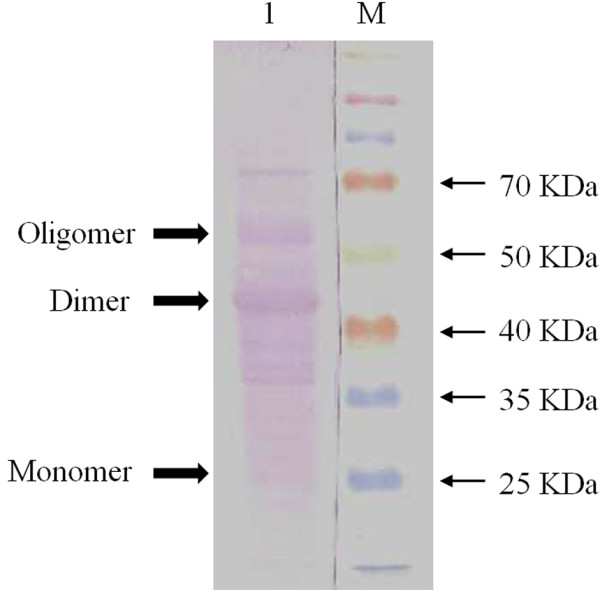
**Western blot of cell disrupted samples using optimized conditions by high pressure homogenizer.** Lane M represents protein marker, lane 1 represents cell disrupted sample. Western blot was performed using the same monoclonal anti-HBsAg as ELISA assay.

It was necessary to scale-up the process with low cost to meet the need for practical requirements. The scale experiments were conducted with the volume of samples increased to 2 L using optimized parameters in the same buffer. The efficiencies and reproducibility of the conditions were evaluated in three batches. The results overall three batches produced a mean value of cell disruption capability of 75.69 ± 2.2% and specific protein release of 29.15 ± 0.63 mg/L. The low standard deviations of the cell disruption capability and specific protein release of HBsAg indicates that the optimized conditions from RSM design were reproducible and capable for process up-scaling. Observation from western blot profile of disrupted sample indicates that HBsAg recovered from the cell disruption process did not suffered from any noticeable damage or degradation and had retained its antigenic properties in generating interaction towards monoclonal anti-HBsAg (Figure 6).

## Discussion

Parameter variables mainly number of passes, biomass concentration and pulse pressure are primary concerns that would affect the outcome of *P. pastoris* cell disruption and the release of HBsAg specific protein. To that extent, optimizations of these parameters are essential for efficient extraction of the protein. With the advantage of statistical design (RSM), it provides an alternative methodology to optimize a particular process by considering the mutual interactions among the factors and to give an estimate of the combined effects of these factors on the final results, which could lead to simplification of process optimization and cheaper production costs. In addition, significant factors that influence the responses to the greatest extent could also be determined.

Among the three significant factors tested, pulse pressure gave the most significant effect compared to that of number of passes and biomass concentration in both cell disruption capability and in release of HBsAg. This indicates that pulse pressure was very important for maintaining the optimal level in the recovery of HBsAg while achieving the best possible cell disruption capability. Pulse pressure has been widely investigated previously in the improvement of cell disruption involving high pressure homogenizer on various types of microorganism. Cell disruption is a two-step process that involves primary rupture of the cell envelope involved a point break, followed by further breakage of the cell wall and degradation of cellular debris [16]. When pressure is applied, disruption increases with increasing level and in certain cases, complete disruption of the initial cell load is achieved. For instance, Kleinig and Middleberg [34] demonstrated for yeast cells, disruption only occurs beyond the pressure of 560 bar. Donsě et al. [35] pointed out that the linear trend of cell disruption kinetics is dependent on pulse pressure to a certain level. For the homogenization of baker’s yeast, a 4-fold increase in degree of cell disruption was observed at 2,500 bar as compared to 500 bar. This pressure dependence were due to the difference in cell membrane and cell wall properties especially for yeast cells, which are considerably harder to disrupt due to its overall very thick and resistant cell wall structure [35,36]. In this matter, Harrison et al. [37] reviewed that yeasts are harder to disrupt as compared to Gram positive and negative bacteria. From Figure 2 B and C, it is evident that experimental data reported in response surface plot as a function of the pressure effect seem to fit well by a straight line suggesting a first order of kinetics for cell disruption capability. In this case, CDR depends linearly on the applied pulse pressure of homogenization regardless of the influence from number of passes and biomass concentration. It was noticed for multi pass homogenization, cell disruption efficiency reduces after each pass tending towards a plateau of an asymptotic value of disruption [35]. This can be attributed to the natural distribution of individual cell resistance to pressure, since the most resistant cells of the initial microbial population are likely to survive the pulses of pressure applied. Therefore, when all the weakest cells are destroyed, no further disruption would occur, even if the number of passes increases [38]. In this case, we supposed that the lack of significance changes in CDR under the determined range was due to the achieved plateau level even at the lowest number of pass for the mentioned pulse pressure range. A threshold value for multiple number of passes effect exists, above which effectiveness of homogenizer process would not be seen. In spite of that, our experimental data appear to be in conflict with the results reported in the literature. Several authors indeed supported the hypothesis that in number of passes treatment, each pass is additive. Each pass would cause the same rate of cell disruption [14,39]. It was observed that successive rounds of high-pressure homogenisation have an additive effect on viability reduction of *Yersinia enterocolitica* and *Staphylococcus aureus*[39].

Also in this case, our results are in agreement with the findings from several authors in that the cell has no discernible influence on cell disruption efficiency over a wide range of cell concentrations [19,40]. For yeast disruption process, it was determined that the process was independent from effects of biomass concentration at a range below 750 g/L [19]. In addition, Van Hee et al. [41] further concluded that biomass concentration does not have a significant effect on cell rupture in the concentration range of 0.06-115 g/L for high pressure homogenizer. It is not unexpected as the main factor in determining the kinetics of cell disruption is the natural distribution of individual cell resistance to high pressure homogenization rather than cell concentration [16]. It is evident that the biomass concentration used in the experiments was in the mentioned ranges, thus the observation appears to be valid. Instead, for the combinative effects on number of passes and biomass concentration, it leads to a clearer trend of dependency. At a fixed pulse pressure, the lower the biomass concentration and number of passes, the higher the cell disruption capability was achieved (Figure 2A). This behaviour could more accurately ascribe the conditions observed in the previous effect of number of passes, at which weaker or more sensitive cells are selectively disrupted in initial few passes below threshold value. Subsequent passes would treat more resistant cells, resulting in lower cell disruption capability [35]. In another approach, Peleg and Cole [42] suggests the hypothesis that cell disruption capability is derived from the cumulative form of resistances or sensitivities distribution within cell population. This approach takes into account of the coexistence of subpopulation which is more resistant to stress or protected by several factors, such as dead cells or secondary product of cellular metabolism. It is also worth noting that the combination effects of number of passes and biomass concentration enhanced the cell disruption capability achieving a higher CDR rate approximately with 18 times number of passes and 6.72 g/L biomass concentration. Nonetheless, the influence was minor in the presence of the pulse pressure effect.

A different behaviour was observed in the release of specific protein from cell disruption process, with the significant factors involving not only pulse pressure but also number of passes and biomass concentration. From Figure 3, all three graphs produce a ‘mountain shaped’ response surface plots which implicates a co-dependency among the variables. Pulse pressure under the influences of number of passes and biomass concentration was observed to enhance specific protein release up to a certain level, above which the release of specific protein curve level off to a decreasing value (Figure 3 B and C). In the literature numerous examples of protein release curves under the influence of pulse pressure which are not governed by the first order of kinetics and gave rise to a non-linear behaviour were reported [19,37]. For example, Keshavarz et al. [42] observed that the release of specific protein alcohol dehydrogenase (ADH) increased to a curvature level accordance to pulse pressure from cell disruption of *Rhizopus nigricans*. Results in support of decrease in the release of specific protein after threshold saturation in the increase of pulse pressure were also reported. Duerre and Ribi [43] concluded that a pressure range of 1,034 bar was sufficient for maximum cell disruption and speculated that degradation of protein can occur when cell disruption was performed at very high pressure range (≥1,734 bar). Ho et al. [42] speculated that the decrease in specific protein release might due to breakage or degradation of the specific protein resulting in ruptured, degraded or unformed particles that has a loss of antigenicity from the samples obtained. In another instance, study performed by Lovitt et al. [20] has shown that maximum specific activity was achieved at pulse pressure of 1,000 bar disruption from baker’s yeast. At higher pressures, up to 2,400 bar, enzyme activity released fell progressively. The observations are consistent with greater quantities of protein becoming solubilized at highest operating pressures but that the specific protein levels in solution remained stagnant or even decline at very high pressures. At the same time the protein released rose rapidly as the operating pressure increased to 1,380 bar, until 2,750 bar where over 85% of the total protein present had been released into a soluble form, thus diluting the specific protein [20]. On the basis of this assumption, it would explain the results obtained for experimental run 7–8 and 14. Under the influence of high pressure range (≥ 1,000 bar), high total soluble protein was released while low specific protein was detected, resulting in low selective product recovery (Figure 4). On the other hand, the loss or decrease in specific protein release could be caused by foam formation during homogenization where large fraction of the specific protein were contained in the foam that was not included in the samples leading to inaccurate sampling [41]. However, this is unlikely as the foam formation in our experiments is only of a minute amount compared to the volume used in the experiment runs. Nevertheless, strong reactivity signal was detected in samples derived from pulse pressure of 1,000 bar and at a biomass of 7.70 g/L. Meanwhile, several studies have reported that increase in pressure above a certain range would result in micronisation of cell debris [40,44,45] and that higher protein release was observed due to the release of insoluble protein complex, peptides, glycopeptides and amino acids [46]. This is coherent with our experimental data, where higher ratio of selective product recovery was achieved in lower pressure range (≤600 bar) in comparison to higher pressure range (≥1,000 bar) due to higher total soluble protein released.

Based on the kinetic-rate law model reported by Hetherington et al. [19] and observations made by Donsě et al. [35], it is possible to predict that the release of specific protein is dependent on the coherent influences of number of passes and pulse pressure. On the basis of such assumption, increasing either one factor would result in the same outcome of protein released and regulation in the increase of both factors would dramatically enhance the release of specific protein to a certain level. From Figure 3B, it is clear that our result is in agreement with the findings in the literature that indeed, additional passes are required for better release of specific protein corresponding to pulse pressure used [17,19,22,46]. In general, a single pass system is thought to be best for the product; however, at a given pressure, multiple passes may be beneficial for further downstream processing depending on the location of the specific protein in the cell. The rate of release can be faster than, equal to, or slower than the overall protein release [17,21,29]. It has to be pointed out that the disruption of cells to a state where membrane associated specific proteins are released are notably to be more difficult. For example, a membrane associated enzyme cytochrome oxidase from *Pseudomonas aeruginosa* would require 3 times or more passes compared to unbound intracellular enzymes that could normally be released in a single pass [29]. In our case, HBsAg produced from *P.pastoris* are membrane associated protein [47] which suggests for the reason of relatively higher number of passes. In addition, difficulty of disruption was also encountered for the difference in growth phase and culture medium. It has been reported that stationary phase cells [16,24,48] and cell grown in complex medium containing yeast extract are more difficult to disrupt [16]. Considerably, similar number of passes (20 times) was also observed to be used in producing efficient breakdown of nano-particles using the Avestin homogenizer [49] while noticeably much more passes (3,000 times) was used in cell disruption of yeast cells prior to purification [50]. Further increase in the number of passes showed a different behaviour for the release of specific protein, with a reduction in the release of HBsAg was observed. Data reported in the literature from cell disruption using high pressure homogenizers in extraction of intracellular material postulated that intracellular material released is not susceptible to shear damage in a significant degree [22,27,45]. Losses are normally due to susceptibility to degradation or inactivation by other factors such as proteases [45]. However, considering that these data were based on low number of passes (1–5 times) this explanation would not properly represent the observations made in this study. Conversely, membrane associated proteins and multi-enzyme complexes were reported to be shear sensitive. For example, in the isolation of a multi-enzyme membrane-associated complex, alkane hydroxylase by homogenization of *Pseudomonas putida* was reported to have badly damaged the enzyme [51]. In another instance, Augenstein et al. [52] concluded that in the release of shear sensitive specific protein from *Bacillus brevis*, degradation of the specific protein occurs beyond a certain number of passes depending on pulse pressure.

Biomass concentration in the combination with pulse pressure and number of passes was shown to influence the outcome of specific protein (Figure 3 A and C). The extent of initial load contributes to the increase and decrease in specific protein release in both cases. Under the influence of number of passes, Save et al. [53] demonstrated that protein release are only 7 times even though cell concentration was increased to 100 times. Thus, it was speculated that the number of passes in protein release are dependent on biomass concentration and dilution rate of the sample. Mosqueira et al. [54] explained that the viscosity of the whole cell suspension is a function of biomass concentration which is an important factor in determining the increase and decrease of specific protein release [40,55]. At a fixed pressure, repeated passes through the homogenizer gradually increases the viscosity of the homogenate, with the extent of the increase depending on the initial biomass concentration. This phenomenon is thought to occur due to cell debris fragmentation and the release of intracellular soluble components, including protein, nucleic acids and polysaccharides which will rise concomitant with increased number of passes [54]. In another instance, Shamlou et al. [56] highlighted that the direct collision between the cells and the walls of the valve involves a maximum impaction force that would only occur in low viscosity, which is an important factor in the disruption of yeast cells [16].

Some studies have shown degradation of high molecular weight compounds subjected to strong shearing forces in a viscous flow [36,57]. Floury et al. [57] found that frictional forces encountered by the high fluid viscosity during high-pressure homogenization induced irreversible degradation of long molecules. Undeniably, there are several authors that supported the hypothesis of which cell disruption under high pressures is independent of biomass concentration [19,37]. Additionally, a non-linear correlation between cell disruption capability and the specific protein release was observed for cell disruption process. This might be attributed to the influences of the effects from the variables studied. A similar condition was Foster [17] in the study on the effects of biomass concentration where increasing cell concentration was found to reduce cell disruption efficiency measured in optical density. However, on assaying the product release, it was found that product concentration actually increased up to 10-fold. Thus, it was concluded that measurement on cell disruption capability alone would not sufficiency evaluate disruption efficiency. Further studies and a careful analysis must be performed in order to clarify the relationship that the cell disruption capability exerts on the release of specific protein.

Also in our case, it is important to address the temperature rise in high pressure homogenization; as a significant rise would cause detrimental effects on process samples. Notably, energy generated from high pressure homogenizers are typically dissipated as heating of the process fluid [29]. The homogenization experiments were performed at an initial temperature suspension ranging from 4-20°C with the aim to exclude the possible thermal effects. During the cell disruption process with increased volume of 2 L, temperature was seen to rise from an initial 4°C start-up. However, it was able to be maintained at ± 20°C throughout the 20 passes with the integrated heat exchanger attached to water bath for the Avestin homogenizer. The temperature maintained was well below temperature zone where heat inactivation or protein degradation can become involved. Wuytack et al. [39] demonstrated that temperature was able to be maintained at ± 18°C during cell disruption process at 3,000 bar using an Avestin homogenizer and is consistent with other high pressure homogenizers where prolong duration of cell disruption process at 1,750 bar for 1 h may still be sustain a temperature of ± 14°C [20]. Interestingly, it was observed that further up-scaling have considerable little effect on the results obtained from the process samples. HBsAg released was demonstrated to have retained its antigenic properties (Figure 6). This was however not unexpected, as the same parameters were used in the up-scaling process with the only exception of volume difference. This is in agreement with the findings of several authors that reported for a fixed pressure and number of passes through the homogenizer, percentage of protein release is unaffected by the type and the scale of operation [28,35]. Under the conditions explored here, the recovery of HBsAg could be performed with different conditions depending on the requirements of the objectives. For instance, high selective product recovery would be generally preferred for further downstream processing as it reduces the impurities in chromatography system and also easier for centrifugation and dead end filtration as the cells are still intact but at a cost of lower specific protein release.

## Conclusion

In this work, the main factors influencing cell disruption of *P. pastoris* by high pressure homogenizer were identified and optimized using RSM. The model equation generated on cell disruption was found adequate to determine the significant factors and its interactions among the process variables and the optimum conditions for cell disruption process. Cell disruption capability was determined to be highly dependent on pulse pressure with no significant effects being seen from the influence of number of passes and biomass concentration. Whereas for the release of specific protein, co-dependency was observed amongst the variables studied. The optimum operating conditions for *P. pastoris* cell disruption process to achieve maximum specific protein release of HBsAg were with number of passes at 20 times, 7.70 g/L of biomass concentration and pulse pressure at 1,029 bar. Cell disruption capability (75.68% CDR) and specific protein release of HBsAg (29.20 mg/L) using optimized cell disruption strategy was 2 times and 4 times higher than that of glass bead cell disruption respectively. Practical conclusions from these observations are that more efficient cell disruption and release of specific protein could be possible with combined process variables and it was possible to conduct cell disruption experiments in a scaled-down homogenizer and confidently applying the results to larger scale units which saves considerable volume of material required to be processed. These findings open up a promising alternative that can help to overcome the problems or costs associated with protein extraction and purification from *P. pastoris* cells in up-scaled production of HBsAg fermentations and downstream processing. This will aid in the production of a more affordable vaccine or for diagnostic kit development which is considered an asset to resource poor and medium level countries.

## Methods

### Fermentation of culture

*P. pastoris* GS115 cells with plasmid pPIC3.5K-HBsAg capable of producing recombinant HBsAg was cultured in BMGY medium (containing 200 μg/mL Geneticin) with shake flask method (Unpublished data). Under the control of *AOXI* promoter, the culture was incubated at 30°C under rigorous shaking at 250 rpm in a shaking incubator. When the biomass concentration reached constant optimal density (OD_600_ ~26), the protein expression was induced with the transfer of *P. pastoris* cells to BMMY medium followed by the daily addition of methanol at a final concentration of 1%. The culture was further incubated for 3 days at 250 rpm at 30°C. *P. pastoris* cells were harvested thereafter by centrifugation at 2,860 x g, for 10 min at 4°C. Prior to cell disruption, the cell pellet was washed with distilled water and centrifuged again at 2,860 x g for 15 min at 4°C. The washing process was repeated twice.

### Glass bead disruption

*P. pastoris* cells containing the recombinant HBsAg protein were subjected to glass bead cell disruption. After the washing process, cells were re-suspended in cell breaking buffer (50 mM sodium phosphate, pH 7.4; 1 mM phenylmethylsulfonyl fluoride (PMSF); 1 mM EDTA and 5% glycerol). Biomass concentration was measured in wet cell weight (WCW) correlating to DCW standard curve. 1 g/L WCW correlates to 0.08g/L DCW. Thereafter, acid washed glass beads (0.5 mm diameter) measured in same weight was then added into the mixture by displacement. The suspension was then vortexed at maximum speed for 1 min with incubation intervals on ice for another 1 min for a total of 20 times. Thereafter, mixture was centrifuged at 2,860 x g for 10 min at 4°C. Supernatant was then separated and transferred to a new centrifuge tube for storage at −20°C until further use.

### High pressure cell disruption

The disruption of yeast cells was carried out using a high pressure homogenizer. Cells were harvested and accumulated from fermentation culture under shake flasks conditions of media using the same medium of inoculum by centrifugation (2,860 x g, 10 min, 4°C), followed by two washes with of 20 mM Tris–HCl buffer (pH 7.0). The appropriate concentration of biomass was measured accordingly in WCW correlating to DCW standard curve and was passed through the homogenizer connected to shell and tube heat exchanger. For temperature control (maintained between 4 to 20°C), cooling water was circulated into the tube side of the heat exchanger. The temperature of the samples thus remained below the temperature zone where heat inactivation can become involved. The cell suspension was then disrupted for a predetermined number of passes (determined as total solution to have gone through the cell disruptor as one pass) with a variation of cell biomass concentration using different pulse pressure as specified in Table 1.

### Experimental design and optimization using RSM

RSM was used to optimize the cell disruption strategy for enhancing the release of HBsAg from *P. pastoris* cells. Number of passes, cell concentration and pulse pressure were regarded as three independent variables whereas cell disruption capability measured as CDR (%) and specific protein release of HBsAg, measured as amount of concentration (mg/L), was considered as response. CCD with rotatable design with five levels was performed. A set of 20 experiments was carried out as calculated in Equation 3 [31].

(3) $$\begin{array}{l} \text{The total number of experimental combinations}\\ \;\text{in CCD}=2^{\text{k}}+2\text{k}+\eta 0 \end{array}$$

where k is the number of variables and Î·0 is the number of repetitions at the central point. For the three factors, this design was made up of a full 2^3^ factorial design with its eight points augmented with six replications of the centre points (all factors at level 0) was used to fit the second-order-response surface. The range and levels of experimental variables investigated are presented in Table 3. The central values (zero level) chosen for the experimental design were: number of passes at 20 times, cell concentration 7.76 g/L DCW and pulse pressure of 1,000 bar. The central values (zero levels) are chosen based on results of previous literature [19,28,45,58] and preliminary studies (data not shown). Initial experiments for cell disruption involving the number of passes (1 to 40 passes), cell concentration (0.08 to 19.41 g/L) and pulse pressure (60 to 1,400 bar) were performed using one-factor-at-one-time strategy. RSM methodology allows the modelling of a second-order equation that describes the process. CDR and specific protein release of HBsAg expressed protein was analysed by multiple regression to fit Equation 4 [31]:

(4) $$\begin{array}{ll} \text{Y} & =\chi_{1}-\chi_{2}\text{A}+\chi_{3}\text{B}\\ & \;+\chi_{4}\text{C}-\chi_{5}{\text{A}}^{2}-\chi_{6}{\text{B}}^{2}-\chi_{7}{\text{C}}^{2}-\chi_{8}\text{AB}-\chi_{9}\text{AC}\\ & \;+\chi_{10}\text{BC} \end{array}$$

where Y is HBsAg concentration; χ_n_ is the constant values; A represents number of passes, B represents cell concentration and C represents pulse pressure. The experimental plan along with the results is presented in Table 1.

The response surface and contour plots were generated for different interaction of any two independent variables, while holding the value of third variable as constant at the central level. Such three-dimensional surfaces could give accurate geometrical representation and provide useful information about the behaviour of the system within the experimental design. The optimization of the cell disruption process was aimed at finding the levels of independent variables (number of passes, cell concentration, pulse pressure), which would give maximum cell disruption capability and HBsAg release activity. The optimum values of the selected variables were obtained by solving the regression equation. RSM was applied to the experimental data using a commercial statistical package (Design Expert ver. 6.0.6, Stat-Ease, Inc., Minneapolis, MN, USA) for the generation of response surface and contour plots and optimization of process variables.

### Sodium dodecyl sulfate-polyacrylamide gel electrophoresis (SDS-PAGE)

The presence of HBsAg was analysed with 12% SDS-PAGE and stained with Coomassie Brilliant Blue R-250 [59]. Electrophoresis was performed at 32 mA for 90 min using the Mini Protean 3 apparatus (Bio-Rad, USA).

### Western Blot

After appropriate dilution with 1X SDS sample disruption buffer, approximately 10 μL of the total extract prepared as described above was loaded onto a SDS-PAGE gel. After electrophoresis, the proteins were transferred to a nitrocellulose membrane. HBsAg was detected using HBsAg primary monoclonal antibody and goat anti-mouse secondary antibody IgG HRP. Finally, the signals were developed using the Western blotting ABTS substrate [10].

### Protein quantification

Protein concentration was determined according to Bradford’s method with slight modification [60]. Briefly, 96-wells microplate was applied as the reaction well and the absorbance was measured by microplate reader (Sunrise) controlled by the Magellan 4.0 PC data analysis software. The amount of protein was calibrated against bovine serum albumin (BSA) as reference standard. Multi-samples were measured in the same microplate and the results were reliable (the relative coefficient (R^2^) of calibration curve of reference protein was above 0.99).

### Enzyme-linked immunosorbent assay (ELISA)

ELISA assay for the detection HBsAg protein was used according to the instructions given by the manufacturer (SURASE-B96 ELISA kit specific for HBsAg detection, General Biologicals Corp, Taiwan). Briefly, proteins released from cell disruption were added into 96 well plates at a volume of 50 μL followed by the addition of another 50 μL of monoclonal anti-HBsAg antibody conjugated to horseradish peroxidase (HRP). The mixture was then incubated for a period of 80 mins at 37°C and thereafter, the well containing the mixture was washed with washing (phosphate buffered saline (PBS), Tween-20) buffer for a total of 3 times at a volume of 200 μL with 30 sec waiting period each time. The plate was then dried and 100 μL of 3, 3', 5, 5'-tetramethylbenzidine (TMB) (0.6 mg/mL TMB, Citrate acid, 0.03% hydrogen peroxide (H_2_O_2_)) substrate was added to each well prior to incubation for 30 mins at room temperature. Addition of 2N Sulphuric acid (H_2_SO_4_) of a volume of 100 μL was performed thereafter before subjecting to ELISA reader (Tecan, Sunrise, Melbourne, Australia) for result observation with a reading wavelength of 450nm and a reference wavelength of 650 nm. To generate a standard curve, a series of dilution containing 0 to 250 ng of HBsAg (Biodesign International, USA) was included in each assay [10]. The amount of HBsAg was then calibrated against standard curve and defined as specific protein release. Selective product recovery, defined as the ratio of specific protein release to total protein release from cell disruption is calculated as follows (Equation 5) [27]:

(5) $$\begin{array}{l} \text{Selective product recovery}\;\left(\text{mg}/\text{g}\right)\\ \;=\frac{\text{specific protein release}\;\left(\text{mg}/\text{L}\right)}{\text{total protein release}\;\left(\text{g}/\text{L}\right)} \end{array}$$

The results obtained were in triplicates at an absorbance value of 450 nm with 650 nm as reference wave length.

### Measurement of CDR

CDR was determined as described previously by using a visible spectrophotometer under a wavelength of 600 nm (Biophotometer, Eppendorf, Germany) by measuring of intact cell density (ICD) before and after passing through samples through high pressure homogenizer [20,61]. Calculation is based in percentage based on Equation 6.

(6) $$\text{CDR}\left(\%\right)=100-\left(\frac{{\text{ICD}}_{\text{after}}\times 100}{{\text{ICD}}_{\text{before}}}\right)$$

## Abbreviations

HBsAg: Hepatitis B surface antigen; CCD: Central composite design; WCW: Wet cell weight; CDR: Cell disruption rate; RSM: Research surface methodology; OD: Optical density; ICD: Intact cell density; G6PDH: Glucose-6-phosphate dehydrogenase; DF: Degrees of freedom.

## Competing interests

The authors declare that they have no competing interests.

## Authors’ contribution

YJT contributed towards all the molecular biology works, cell disruption experiments, acquisition of data and data analysis and interpretation of the study. ZNA, JST and MAR involved in drafting of initial experiments one-factor-at-a-time strategy followed by conception and design of RSM and later the manuscript. MAML and ARB conceived of the study, and participated in its design and coordination and helped to draft the manuscript. All authors read and approved the final manuscript.

## Authors’ information

YJT and MAR are PhD students while JST is a post doctorate at Laboratory of Immunotherapeutic and Vaccine Technology (LIVES). ZNA is an associate professor while MAML and ARB are professors at Faculty Veterinary Medicine.

## References

[B1] Torbenson M Thomas DL Occult hepatitis B Lancet Infect Dis 2002 2 8 479 486 10.1016/S1473-3099(02)00345-6 12150847

[B2] Sunil Kumar G Ganapathi T Srinivas L Revathi C Bapat V Expression of hepatitis B surface antigen in potato hairy roots Plant Sci 2006 170 5 918 925 10.1016/j.plantsci.2005.12.015

[B3] Sung J Hepatitis B virus eradication strategy for Asia Vaccine 1990 8 S81 S85 2139290 10.1016/0264-410x(90)90227-d

[B4] Dehesa-Violante M Nuñez-Nateras R Epidemiology of hepatitis virus B and C Arch Med Res 2007 38 6 606 611 10.1016/j.arcmed.2007.03.001 17613351

[B5] Lai CL Ratziu V Yuen MF Poynard T Viral hepatitis B Lancet 2003 362 9401 2089 2094 10.1016/S0140-6736(03)15108-2 14697813

[B6] Bruss V Ganem D Mutational analysis of hepatitis B surface antigen particle assembly and secretion J Virol 1991 65 7 3813 3820 2041095 10.1128/jvi.65.7.3813-3820.1991 PMC241412

[B7] Shiosaki K Takata K Nishimura S Mizokami H Matsubara K Production of hepatitis B virion-like particles in yeast Gene 1991 106 2 143 10.1016/0378-1119(91)90193-F 1937046

[B8] Fujisawa Y Ito Y Sasada R Ono Y Igarashi K Marumoto R Kikuchi M Sugino Y Direct expression of hepatitis B surface antigen gene in E coli Nucleic Acids Res 1983 11 11 3581 3591 10.1093/nar/11.11.3581 6304635 PMC325988

[B9] Hitzeman RA Chen CY Hagie FE Patzer EJ Liu CC Estell DA Miller JV Yaffe A Kleid DG Levinson AD Expression of hepatitis B virus surface antigen in yeast Nucleic Acids Res 1983 11 9 2745 2763 10.1093/nar/11.9.2745 6344021 PMC325921

[B10] Morvarid A Zeenathul N Tam Y Zuridah H Mohd-azmi M Azizon B Effect of glycerol feed in methanol induction phase for hepatitis B surface antigen expression in Pichia pastoris strain KM71 Pertanika J Sci & Technol 2012 20 1 31 42

[B11] Hardy E Martínez E Diago D Díaz R González D Herrera L Large-scale production of recombinant hepatitis B surface antigen from *Pichia pastoris* J Biotechnol 2000 77 2 157 167 2 10682276 10.1016/s0168-1656(99)00201-1

[B12] Michel ML Sobczak E Malpièce Y Tiollais P Streeck RE Expression of amplified hepatitis B virus surface antigen genes in Chinese hamster ovary cells Nat Biotechnol 1985 3 6 561 566 10.1038/nbt0685-561

[B13] Sunil Kumar G Ganapathi T Revathi C Prasad K Bapat V Expression of hepatitis B surface antigen in tobacco cell suspension cultures Protein Expr Purif 2003 32 1 10 17 10.1016/j.pep.2003.07.004 14680934

[B14] Kleinig AR Middelberg APJ The correlation of cell disruption with homogenizer valve pressure gradient determined by computational fluid dynamics Chem Eng Sci 1996 51 23 5103 5110 10.1016/S0009-2509(96)00354-5

[B15] Fish NM Lilly MD The interactions between fermentation and protein recovery Nat Biotechnol 1984 2 7 623 627 10.1038/nbt0784-623

[B16] Middelberg APJ Process-scale disruption of microorganisms Biotechnol Adv 1995 13 3 491 551 10.1016/0734-9750(95)02007-P 14536098

[B17] Foster D Optimizing recombinant product recovery through improvements in cell-disruption technologies Curr Opin Biotechnol 1995 6 5 523 526 10.1016/0958-1669(95)80086-7

[B18] Schütte H Kula M-R Cell disruption and isolation of non-secreted products. In: Biotechnology Set 2008 Weinheim, Germany: Wiley-VCH Verlag GmbH 505 526

[B19] Hetherington PJ Follows M Dunnill P LlLly MD Release of protein from baker’s yeast (*Saccharomyces cerevisiae*) by disruption in an industrial homogenizer Chem Eng Res Des 1971 49 a 142 148

[B20] Lovitt RW Jones M Collins SE Coss GM Yau CP Attouch C Disruption of bakers’ yeast using a disruptor of simple and novel geometry Process Biochem 2000 36 5 415 421 10.1016/S0032-9592(00)00223-5

[B21] Follows M Hetherington PJ Dunnill P Lilly MD Release of enzymes from bakers’ yeast by disruption in an industrial homogenizer Biotechnol Bioeng 1971 13 4 549 560 10.1002/bit.260130408 4944069

[B22] Moore EK Hoare M Dunnill P Disruption of baker’s yeast in a high-pressure homogenizer: new evidence on mechanism Enzyme Microb Technol 1990 12 10 764 770 10.1016/0141-0229(90)90149-K

[B23] Pandolf WD High-pressure homogenization Chem Process 1998 61 3 39 43

[B24] Engler CR Asenjo JA Cell disruption by homogenizer Separation Processes in Biotechnology 1990 9 95 105 1366892

[B25] Diels AMJ De Taeye J Michiels CW Sensitisation of *Escherichia coli* to antibacterial peptides and enzymes by high-pressure homogenisation Int J Food Microbiol 2005 105 2 165 175 10.1016/j.ijfoodmicro.2005.04.027 16126294

[B26] Lovering AL Strynadka NCJ High-resolution structure of the major periplasmic domain from the cell shape-determining filament MreC J Mol Biol 2007 372 4 1034 1044 10.1016/j.jmb.2007.07.022 17707860

[B27] Ramanan RN Tey BT Ling TC Ariff AB Classification of pressure range based on the characterization of *Escherichia coli* cell disruption in high pressure homogenizer Am J Biochem Biotech 2009 5 21 29

[B28] Siddiqi SF TitchenerÃÂ¢-Hooker NJ Shamlou PA High pressure disruption of yeast cells: the use of scale down operations for the prediction of protein release and cell debris size distribution Biotechnol Bioeng 1997 55 4 642 649 10.1002/(SICI)1097-0290(19970820)55:4<642::AID-BIT6>3.0.CO;2-H 18636574

[B29] Chisti Y Moo-Young M Disruption of microbial cells for intracellular products Enzyme Microb Technol 1986 8 4 194 204 10.1016/0141-0229(86)90087-6

[B30] Adinarayana K Ellaiah P Srinivasulu B Bhavani Devi R Adinarayana G Response surface methodological approach to optimize the nutritional parameters for neomycin production by *Streptomyces marinensis* under solid-state fermentation Process Biochem 2003 38 11 1565 1572 10.1016/S0032-9592(03)00057-8

[B31] Tan JS Ramanan RN Azaman SNA Ling TC Shuhaimi M Ariff AB Enhanced interferon-α2b production in periplasmic space of *Escherichia coli* through medium optimization using response surface method Open Biotechnol J 2009 3 117 124

[B32] Sánchez-Romeu J País-Chanfrau JM Pestana-Vila Y López-Larraburo I Masso-Rodríguez Y Linares-Domínguez M Márquez-Perera G Statistical optimization of immunoaffinity purification of hepatitis B surface antigen using response surface methodology Biochem Eng J 2008 38 1 1 8 10.1016/j.bej.2007.05.016

[B33] Li C Bai J Cai Z Ouyang F Optimization of a cultural medium for bacteriocin production by *Lactococcus lactis* using response surface methodology J Biotechnol 2002 93 1 27 34 10.1016/S0168-1656(01)00377-7 11690692

[B34] Kleinig AR Middelberg APJ On the mechanism of microbial cell disruption in high-pressure homogenisation Chem Eng Sci 1998 53 5 891 898 10.1016/S0009-2509(97)00414-4

[B35] Donsě F Ferrari G Lenza E Maresca P Main factors regulating microbial inactivation by high-pressure homogenization: Operating parameters and scale of operation Chem Eng Sci 2009 64 3 520 532 10.1016/j.ces.2008.10.002

[B36] Diels AMJ Michiels CW High-pressure homogenization as a non-thermal technique for the inactivation of microorganisms Crit Rev Microbiol 2006 32 4 201 216 10.1080/10408410601023516 17123905

[B37] Harrison STL Bacterial cell disruption: a key unit operation in the recovery of intracellular products Biotechnol Adv 1991 9 2 217 240 10.1016/0734-9750(91)90005-G 14548738

[B38] Donsì G Ferrari G Maresca P Pulsed high pressure treatment for the inactivation of *Saccharomyces cerevisiae*: the effect of process parameters J Food Eng 2007 78 3 984 990 10.1016/j.jfoodeng.2005.12.042

[B39] Wuytack EY Diels AMJ Michiels CW Bacterial inactivation by high-pressure homogenisation and high hydrostatic pressure Int J Food Microbiol 2002 77 3 205 212 10.1016/S0168-1605(02)00054-5 12160080

[B40] Agerkvist I Enfors SO Characterization of E. coli cell disintegrates from a bead mill and high pressure homogenizers Biotechnol Bioeng 1990 36 11 1083 1089 10.1002/bit.260361102 18595048

[B41] Van Hee P Middelberg APJ Van Der Lans RGJM Van Der Wielen LAM Relation between cell disruption conditions, cell debris particle size, and inclusion body release Biotechnol Bioeng 2004 88 1 100 110 10.1002/bit.20343 15449302

[B42] Peleg M Cole MB Reinterpretation of microbial survival curves Crit Rev Food Sci 1998 38 5 353 380 10.1080/10408699891274246 9704188

[B43] Duerre JA Ribi E Enzymes released from Escherichia coli with the aid of a Servall cell fractionator Appl Microbiol 1963 11 6 467 471 14075044 10.1128/am.11.6.467-471.1963 PMC1058031

[B44] Bailey SM Meagher MM Crossflow microfiltration of recombinant Escherichia coli lysates after high pressure homogenization Papers Biotechnol 1997 56 3 304 310 10.1002/(SICI)1097-0290(19971105)56:3<304::AID-BIT8>3.0.CO;2-N 18636646

[B45] Keshavarz E Bonnerjea J Hoare M Dunnill P Disruption of a fungal organism, Rhizopus nigricans, in a high-pressure homogenizer Enzyme Microb Technol 1990 12 7 494 498 10.1016/0141-0229(90)90064-W

[B46] Limon-Lason J Hoare M Orsborn CB Doyle DJ Dunnill P Reactor properties of a high-speed bead mill for microbial cell rupture Biotechnol Bioeng 1979 21 5 745 774 10.1002/bit.260210503

[B47] Vassileva A Chugh DA Swaminathan S Khanna N Expression of hepatitis B surface antigen in the methylotrophic yeast Pichia pastoris using the GAP promoter J Biotechnol 2001 88 1 21 35 10.1016/S0168-1656(01)00254-1 11377762

[B48] Harrison S Chase H Dennis J The disruption of Alcaligenes eutrophus by high pressure homogenisation: key factors involved in the process Bioseparation 1991 2 3 155 1368082

[B49] Abbasalipourkabir R Salehzadeh A Abdullah R Cytotoxicity effect of solid lipid nanoparticles on human breast cancer cell lines Biotechnology 2011 10 528 533 10.3923/biotech.2011.528.533

[B50] Balasundaram B Harrison STL Influence of the extent of disruption of Bakers’ yeast on protein adsorption in expanded beds J Biotechnol 2008 133 3 360 369 10.1016/j.jbiotec.2007.07.724 17933410

[B51] Fish NM Harbron S Allenby DJ Lilly MD Oxidation of n-alkanes: isolation of alkane hydroxylase from Pseudomonas putida Appl Microbiol Biotechnol 1983 17 1 57 63 10.1007/BF00510573

[B52] Augenstein D Thrasher K Sinskey A Wang D Optimization in the recovery of a labile intracellular enzyme Biotechnol Bioeng 1974 16 11 1433 1447 10.1002/bit.260161102 4441628

[B53] Save S Pandit A Joshi J Microbial cell disruption: role of cavitation Chem Eng J Biochem Eng J 1994 55 3 B67 B72 10.1016/0923-0467(94)06062-2

[B54] Mosqueira F Higgins J Dunnill P Lilly M Characteristics of mechanically disrupted bakers’ yeast in relation to its separation in industrial centrifuges Biotechnol Bioeng 1981 23 2 335 343 10.1002/bit.260230208

[B55] Harrison ST Chase HA Dennis JS The disruption of *Alcaligenes eutrophus* by high pressure homogenisation: key factors involved in the process Bioseparation 1991 2 3 155 166 1368082

[B56] Ayazi Shamlou P Siddiqi S Titchener-Hooker N A physical model of high-pressure disruption of bakers’ yeast cells Chem Eng Sci 1995 50 9 1383 1391 10.1016/0009-2509(94)00475-7

[B57] Floury J Desrumaux A Axelos MAV Legrand J Degradation of methylcellulose during ultra-high pressure homogenisation Food Hydrocolloids 2002 16 1 47 53 10.1016/S0268-005X(01)00039-X

[B58] Heim A Kamionowska U Solecki M The effect of microorganism concentration on yeast cell disruption in a bead mill J Food Eng 2007 83 1 121 128 10.1016/j.jfoodeng.2007.02.047

[B59] Laemmli UK Cleavage of structural proteins during the assembly of the head of bacteriophage T4 Nature 1970 227 5259 680 685 10.1038/227680a0 5432063

[B60] Kruger NJ Walker JM The Bradford Method for Protein Quantitation The Protein Protocols Handbook 2002 Totowa, NJ: Humana Press 15 21

[B61] Ho CW Chew TK Ling TC Kamaruddin S Tan WS Tey BT Efficient mechanical cell disruption of *Escherichia coli* by an ultrasonicator and recovery of intracellular hepatitis B core antigen Process Biochem 2006 41 8 1829 1834 10.1016/j.procbio.2006.03.043

